# An innovative porcine model of pediatric drowning^☆^

**DOI:** 10.1016/j.resplu.2026.101460

**Published:** 2026-08-26

**Authors:** Chelsea Morin, Megan O’Reilly, Tze-Fun Lee, Georg M Schmölzer

**Affiliations:** Centre for the Studies of Asphyxia and Resuscitation, Neonatal Research Unit, Royal Alexandra Hospital, Edmonton, Alberta, Canada; Department of Pediatrics, University of Alberta, Edmonton, Alberta, Canada

**Keywords:** Drowning, Asphyxia, Cardiopulmonary resuscitation, Children, Animal models

## Abstract

Worldwide, children between the ages of 0 and 4 years have the highest rate of drowning incidents. Hypoxia is the most detrimental consequence of drowning incidents. Therefore, restoration of oxygenation, ventilation, and perfusion is of the utmost importance and should be restored as rapidly as possible via adequate airway management and respiratory support during cardiopulmonary resuscitation (CPR). Optimizing the CPR technique used following drowning incidents in children, has the potential to improve survival. There is currently extremely limited scientific knowledge regarding the optimal CPR technique for pediatric drowning victims. Indeed, there is no pediatric animal drowning model currently available, which makes our model unique. Our model lays the foundation for future pre-clinical and clinical studies and will also provide critical scientific knowledge used to guide resuscitation recommendations.

## Introduction

Drowning is defined by the World Health Organization as “the process of experiencing respiratory impairment from submersion/immersion in liquid”.^1^ It remains one of the three leading causes of unintentional pediatric injury worldwide, with the highest incidence among children aged 0–4 years old.^1^, ^2^, ^3^ Globally, drowning is the second leading cause of preventable death and the primary cause of cardiac arrest in children.^1^, ^4^, ^5^ Pathophysiological, drowning results in progressive hypoxia and respiratory acidosis, followed by the development of metabolic acidosis.^6^ If hypoxia persists, cardiac arrest ensues. Although cardiac arrest during drowning is typically secondary to respiratory failure, a primary cardiac event may occasionally precipitate drowning. Shockable rhythms are identified in approximately 2–12% of drowning victims who experience cardiac arrest.^4^ Shorter duration of submersion is the strongest predictor of favorable outcomes after pediatric cardiac arrest from drowning; with submersion time less than 5 min associated with the best prognosis.^4^, ^6^

Morbidity after an initially successful resuscitation can be high, with survivors experiencing unfavorable neurological outcomes due to brain hypoxia.^7^ Therefore, restoration of oxygenation, ventilation, and perfusion is of the utmost importance and should be restored as rapidly as possible via adequate airway management and respiratory support during resuscitation.^8^ The 2024 American Heart Association and American Academy of Pediatrics Focused Update on Resuscitation Following Drowning emphasizes the lack of randomized control trials and the limited evidence-base guiding both resuscitation and post-drowning management.^4^ While over the last twenty years, some building blocks for drowning resuscitation research have been established,^4^, ^9^, ^10^ there is a lack of studies to address the optimal approach for cardiopulmonary resuscitation (CPR) after drowning. A recent systematic review of interventions for resuscitation following drowning reported no studies, which compared Airway-Breathing-Circulation (ABC) versus Circulation-Airway-Breathing (CAB) sequences and only two observational studies comparing compression-only CPR versus standard CPR.^11^ Standard CPR compared to compression-only CPR reduced time to return of spontaneous circulation (ROSC), and improved survival (14.4% vs 11%), and favorable neurological outcome (11% vs. 9%),^11^ suggesting an important role for providing adequate ventilation and oxygenation.

For unresponsive drowning victims with cardiac arrest, the current Pediatric Advanced Life Support (PALS) recommend CPR using a 15:2 compression-to-ventilation ratio (C:V) with bag and mask ventilation.^8^, ^12^ Once an advanced airway has been established (tracheal intubation), CPR should be performed using continuous chest compressions with asynchronous ventilation (CCaV), using a ventilation rate of 20–30 inflations/min.^8^ However, the approach to optimize coronary and cerebral perfusion while providing adequate ventilation during pediatric CPR are largely extrapolated from studies with adult patients and adult animal studies. Those data may not apply wholly to the pediatric population. We need in-depth, dedicated pediatric resuscitation research using age-appropriate animal models to determine the optimal CPR technique. Consequently, an animal model that reproduces pediatric drowning physiology is invaluable for investigating resuscitation strategies. Here, we describe a pediatric piglet model of drowning-induced hypoxia, which is adapted from our extensive experience using neonatal, infant, and pediatric piglet models of hypoxia-induced cardiac arrest.^13^, ^14^, ^15^ The purpose of this paper is to describe the protocol of the pediatric piglet drowning model, which can be adapted by researchers for further detailed studies to advance our scientific knowledge.

### Piglet model of cardiac arrest

Neonatal, infant, and pediatric piglets share close anatomical and physiological similarities with human neonates and young children, making them ideal translational models for studying cardiac arrest and CPR.^16^, ^17^ Piglets have comparable body size and weight, a similar shaped minimally keeled chest, and can be instrumented using catheters and endotracheal tubes of sizes equivalent to those used in human infants. Importantly, they exhibit similar cardiopulmonary and metabolic responses to asphyxia and resuscitation. These shared features enable realistic modeling of human pathophysiology during hypoxia-induced cardiac arrest and CPR, supporting their use in both neonatal and pediatric resuscitation research (Table 1).

**Table 1:** Advantages of the proposed porcine model.

**Domain**	**Advantages**
Translational Relevance	Piglets share close anatomic and physiological similarities to human infants – comparable airway size, thoracic geometry, and cardiopulmonary responses to hypoxia and resuscitation.
Experimental Control	Allows precise regulation of oxygenation, ventilation, and temperature; avoids uncontrolled environmental variables inherent to full submersion.
Versatility	Easily modified to simulate various drowning types (e.g., freshwater, saltwater, hypothermic).
Safety and Reproducibility	Non-submersion approach prevents contamination of equipment, permits safe defibrillation, and ensures consistent induction of hypoxia.
Comprehensive Monitoring	Enables real-time assessment of cerebral and myocardial perfusion, oxygen delivery, and post-resuscitation recovery.
Post-Resuscitative Research	Allows extended observation to evaluate cardiovascular recovery, neurologic outcomes, and adjunctive therapies.
Ethical Refinement	Incorporates 3Rs principles and the Canadian Council on Animal Care guidelines for scientific procedures framework – minimizing distress, using humane endpoints, and maximizing data yield per animal.

## Methods

### Animal preparation

Mixed-breed piglets are obtained on the day of experimentation from the University of Alberta Swine Research and Technology Center. All procedures are conducted in accordance with the guidelines and approval of the Animal Care and Use Committee (Health Sciences), University of Alberta. The studies are reported following the ARRIVE 2.0 guidelines and registered at preclinicaltrials.eu.^18^

### Inclusion and exclusion criteria

Piglets are included if they are healthy and weigh within the expected range for age (weeks 3–8 of life = 1–2 years of infant age, weight 8–15 kg). Good health indicators include normal body temperature (38.5–39.5 °C), normal and steady weight gain for age with absence of signs of malnutrition, clear/clean eyes, skin/coat, and nasal passages free of lesions/scabs and any abnormal discharge or inflammation, quiet and even respiration free from panting or open-mouth gasping, normal coordinated gait with absence of any physical abnormalities. Animals are excluded if they exhibit congenital anomalies or signs of infection prior to experimental intervention.

### Randomization and blinding

Piglets are randomly allocated to treatment groups using block randomization with variable block sizes, generated through an online randomization program (https://www.randomizer.org). Sequentially numbered, sealed, opaque envelopes containing the group assignments are opened at the time of experimentation. Where feasible, investigators responsible for assessing the timing of cardiac arrest and analyzing outcome data are blinded to group allocation.

### Sample size and power calculation

Sample size estimates are determined a priori based on expected differences in the primary outcome variable and variability observed in prior studies. Calculations are based on 80% power to detect a clinically relevant difference at a two-tailed alpha of 0.05. Implementing statistical method strategies, such as power analysis, to determine the minimum sample size necessary for reliable results enables a reduction in the number of animals needed while still obtaining sufficient data for the research objectives.

### Anesthesia and instrumentation

**Fig. 1:**
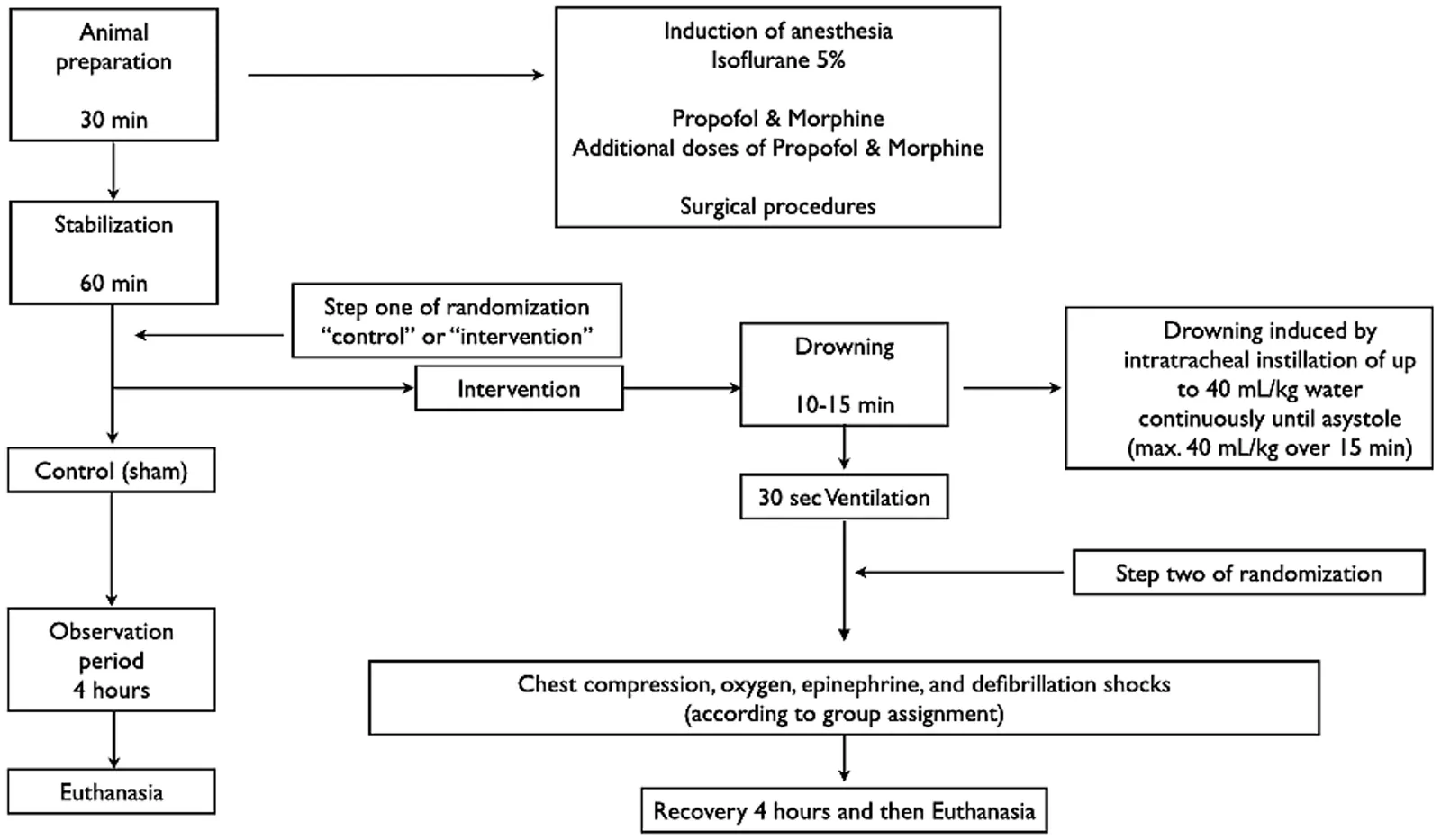
**Study flow chart**.

Premedication with a combined intramuscular dose of ketamine (11 mg/kg) and midazolam (150 µg/kg) is administered to establish sedation prior to anesthesia. Anesthesia is induced with isofluorane gas (titrated from 3% to 5%) delivered at 4 L/min via face mask to initiate anesthesia (Fig. 1).

Electrode leads are placed on the right and left forelimb and the left hindlimb for continuous 3-lead electrocardiogram monitoring, and a pulse oximetry probe is placed on a right forelimb to measure percutaneous oxygen saturation monitoring. Once vascular access is established, a 5 French Argyle® (Klein-Baker Medical Inc. San Antonio, TX) single-lumen catheter is placed in the femoral artery and a 5 French Argyle® (Klein-Baker Medical Inc. San Antonio, TX) double-lumen catheter placed in the femoral vein. Intravenous infusions of morphine (100 µg/kg/h) and propofol (5–10 mg/kg/h) are initiated and titrated to maintain both anesthesia and analgesia. A 5% dextrose solution is infused intravenously at 10 mL/kg/h to maintain hydration and normoglycemia. A real-time ultrasonic flow probe (2 mm; Transonic Systems Inc., Ithaca, NY) is placed around the right common carotid artery to measure cerebral blood flow.

The piglet is intubated with a 4.5 mm uncuffed endotracheal tube with medication port, secured via tracheotomy. Pressure-controlled ventilation (Sechrist Infant Ventilator Model IV-100; Sechrist Industries, Anaheim, CA) is commenced with a respiratory rate of 16–20 breaths/min, peak inflation pressures of 20 cmH_2_O and a positive end-expiratory pressures of 5 cmH_2_O. Fraction of inspired oxygen is adjusted to maintain SpO_2_ between 94% and 100%.

### Respiratory function monitoring

A respiratory function monitor (NM3, Respironics, Philips, Andover, MA) is used to continuously measure tidal volume (*V_T_*), airway pressures, gas flow, and end-tidal CO_2_.^19^, ^20^ The NM3 flow sensor uses a fixed orifice pneumotach to measure the pressure differential across the orifice, which is used to calculate gas flow and derive inspiratory and expiratory *V_T_*. The flow sensor is placed between the endotracheal tube and the ventilation device. V_T_ is calculated by integrating the flow signal. The device accuracy for gas flow is ±0.125 L/min.

### Cerebral perfusion monitoring

Regional cerebral oxygen saturation (crSO_2_) is continuously measured using the INVOS™ Cerebral/Somatic Oximeter Monitor (INVOS 5100, Somanetics Corp., Troy, MI).^21^ The sensor is placed on the right forehead and secured with adhesive wrap and tape; a light-shielding cap is used to minimize ambient interference. The device calculates crSO_2_ as the ratio of oxygenated to total hemoglobin, expressed as a percentage. Values are recorded every second at a sampling rate of 0.13 Hz. The device has a minimum reading limit of 15%, therefore any crSO_2_ measurement lower than 15% will display as 15%.

## Data recording

Continuous monitoring of heart rate, blood pressure (systolic, diastolic, and mean) and percutaneous oxygen saturation are recorded with a Hewlett Packard 78833B patient monitor (Hewlett Packard Co., Palo Alto, CA).

## Recovery

Following surgical instrumentation, piglets are positioned supine and allowed to recover until baseline hemodynamic parameters are stable for at least one hour. Normothermia (38.5–39.5 °C) is maintained using a heating pad and overhead warmer. Normocapnia (35–45 mmHg) is maintained by adjusting ventilator settings. Continuous monitoring and intensive care management of vitals (body temperature, analgesia/anesthesia, hemodynamic and respiratory parameters) enables stabilization during the surgical procedures and during post-surgery recovery. Maintaining tight physiological stability, combined with advanced surgical technique and optimal analgesia/anesthesia ensures minimal procedural distress and improves the overall quality of data obtained. Refinement measures are guided by the Canadian Council on Animal Care (CAAC) framework for scientific procedures.

### Sex differences

In our previous animal studies using newborn piglets we have not observed sex differences,^22^, ^23^ however, any analysis includes exploratory subgroup analysis by sex (male/female) and results are reported separately for males and females to uncover any sex differences.

## Experimental protocol: drowning-induced hypoxia

In this model, drowning is simulated by flooding the lungs with fluid, rather than through full-body submersion (Fig. 1). This approach induces hypoxia by filling the lungs with liquid, reproducing the respiratory and metabolic derangements seen in submersion-induced hypoxia, while remaining practical and controlled in a laboratory environment. Additionally, this technique allows for precise regulation of body temperature, enabling either hypothermic or normothermic conditions to be maintained as part of the experimental design.

To induce drowning, water is instilled through the endotracheal medication port (clamping the endotracheal tube proximal to the medication port) at a rate of up to 60 mL/min. The total instilled volume does not exceed 40 mL/kg. Fluid instillation continues until either the predefined criterion for cardiac arrest is met, or the maximum volume of 40 mL/kg is administered. Cardiac arrest criterion includes a mean arterial blood pressure of ≤23 mmHg and bradycardia (∼<50% of baseline heart rate) confirmed by auscultation of the heartbeat and detection with electrocardiogram monitoring. If cardiac arrest does not occur after the total fluid volume is instilled, the piglet remains in a hypoxic state for a predetermined maximum interval (up to 10 min) before resuscitation is initiated.

Once the predefined cardiac arrest criterion or maximum time interval is reached, resuscitation begins with suctioning through the endotracheal tube. Although this step is not necessarily representative of clinical drowning scenarios, it prevents fluid from entering the ventilator circuit and allows for standardized initiation of resuscitative efforts. The brief delay introduced by this suctioning process approximates the real-world delay often encountered between water rescue, recognition of cardiac arrest, and initiation of CPR. Table 2 provides an example of the changes in hemodynamic and blood gas parameters before and after inducing drowning in this model, using 0.9% sodium chloride at room temperature. Hypoxic insult may be variable between animals even with the same drowning procedure. Therefore, it is recommended to evaluate hypoxia severity based off the following blood gas values, which are representative of a severe insult: PaO_2_ < 40 mmHg, PaCO_2_ > 45 mmHg, pH < 7.35, lactate > 4.0 mmol/L, base excess < −6.0 mmol/L. In our described model, we demonstrate a significant change in the abovementioned blood gas values from before to immediately after fluid instillation (Table 2), which is indicative of drowning-induced hypoxia.

**Table 2:** Demographics, hemodynamic and blood gas changes before and after drowning.

	**Demographics**	**Before drowning**	**After drowning/cardiac arrest**	***P* values**
Age (days)	23.5 (21–25)			
Weight (kg)	7.8 (7.5–8.3)			
Sex	6 male, 4 female			
Heart rate (bpm)		144 (131–158)	73 (69–83)	*p* < 0.001
Mean arterial pressure (mmHg)		75 (70–87)	23 (22–23)	*p* < 0.001
Carotid flow (ml/min)		58 (53–85)	6 (3–10)	*p* < 0.001
Cerebral oxygenation (%)		52 (40–55)	15 (15–15)#	*p* < 0.001
pH		7.46 (7.39–7.49)	7.06 (6.99–7.12)	*p* < 0.001
PaO_2_		98 (90–116)	7 (5–7)	*p* < 0.001
PaCO_2_		34 (33–36)	70 (65–77)	*p* < 0.001
Base access		0.4 (−1.3 to 1.5)	−11.3 (−14.1 to −7.0)	*p* < 0.001
Lactate		2.9 (2.2–3.8)	9.9 (7.2–13.9)	*p* < 0.001

Data are presented as median (IQR) from 10 pigs; Drowning fluid: room temperature 0.9% sodium chloride; Before drowning time point is at the end of the stabilization period, immediately prior to commencing fluid instillation; After drowning/cardiac arrest time point is when the predefined cardiac arrest criterion or maximum time interval is reached following fluid instillation, immediately prior to commencing CPR.

# Note: the device measuring cerebral oxygenation has a minimum reading limit of 15%.

## Potential experimental studies

The described pediatric piglet drowning model provides a flexible platform for investigating a wide range of resuscitation strategies, pathophysiologic mechanisms, and therapeutic interventions relevant to pediatric cardiac arrest secondary to drowning. This model enables systematic evaluation of resuscitation techniques, pharmacologic therapies, and environmental variables under controlled experimental conditions.

This model enables systematic comparison of resuscitation strategies, including ABC versus CAB sequences, standard PALS CPR versus alternative CPR techniques, and varying oxygen concentrations or ventilation modes during CPR. It also supports evaluation of epinephrine versus vasopressin, normothermia versus therapeutic hypothermia, and the influence of different drowning media such as saltwater, freshwater, or pool water. Together, these experiments allow comprehensive investigation of physiologic, pharmacologic, and environmental factors influencing outcomes after pediatric drowning-induced cardiac arrest. Beyond resuscitation parameters, this model enables measurement of cerebral and myocardial perfusion, oxygen delivery, and mitochondrial function, hemodynamic stability and recovery kinetics post-return of spontaneous circulation, biomarkers of injury and inflammation, and long-term neurophysiologic and histopathologic outcomes in survival experiments. This model also provides a means for studying post-return of spontaneous circulation care, such as surfactant administration or therapeutic hypothermia. Given the wide range of potential experimental studies that may arise from this model, it is imperative that the Utstein-style guidelines for reporting of laboratory CPR research are utilized,^24^ to enhance reporting consistency, transparency, and scientific communication.

## Advantages

There are numerous advantages to this non-submersion piglet model of pediatric drowning. The model can be easily modified to replicate a wide range of drowning scenarios, including such as freshwater, saltwater, and hypothermic drowning. It allows for controlled induction of hypoxia while maintaining experimental safety and reproducibility. Importantly, the setup enables prolonged post-resuscitative monitoring, facilitating studies on cardiovascular recovery, neurologic outcomes, and targeted post-arrest therapies. Furthermore, the non-submersion approach permits safe administration of electrical shocks during resuscitation, supporting comprehensive evaluation of both non-shockable and shockable cardiac arrest rhythms.

## Limitations and refinement

While this model offers high translational relevance, several limitations must be acknowledged. The controlled laboratory environment does not fully replicate the variability of real-world drowning events, such as differences in submersion duration, temperature, and contamination. Anesthetic use may alter physiological responses compared to spontaneous drowning in awake children, and the sample size in preclinical models may limit generalizability. Nonetheless, the model has been refined to minimize animal use and distress, employing rigorous monitoring, humane endpoints, and adherence to the 3Rs principle (Replacement, Reduction, and Refinement).^18^, ^25^ These refinements ensure animal welfare while preserving the validity and reproducibility of physiologic and resuscitation data.

## CRediT authorship contribution statement

**Chelsea Morin:** Writing – review & editing, Writing – original draft, Methodology, Investigation, Funding acquisition, Formal analysis, Data curation, Conceptualization. **Megan O’Reilly:** Writing – review & editing, Resources, Project administration, Methodology, Investigation, Formal analysis, Data curation, Conceptualization. **Tze-Fun Lee:** Writing – review & editing, Validation, Project administration, Methodology, Investigation, Formal analysis, Data curation, Conceptualization. **Georg M Schmölzer:** Writing – review & editing, Validation, Supervision, Resources, Methodology, Investigation, Funding acquisition, Formal analysis, Data curation, Conceptualization.

## Declaration of competing interest

CM, MOR, TFL, and GMS have no competing interests.
